# *Ingolfiella maldivensis* sp. n. (Crustacea, Amphipoda, Ingolfiellidae) from coral reef sand off Magoodhoo island, Maldives

**DOI:** 10.3897/zookeys.449.8544

**Published:** 2014-10-22

**Authors:** Ronald Vonk, Damiá Jaume

**Affiliations:** 1 Naturalis Biodiversity Center, P. O. Box 9517, 2300 RA Leiden, The Netherlands; 2 Institute for Biodiversity and Ecosystem Dynamics, University of Amsterdam, Amsterdam 1098 XH, The Netherlands; 3 Instituto Mediterráneo de Estudios Avanzados, IMEDEA (CSIC-UIB), C/ Miquel Marquès 21, 07190 Esporles, Balearic Islands, Spain

**Keywords:** Taxonomy, sublittoral, interstitial habitat, dive sampling, atolls, Indian Ocean

## Abstract

A new species of marine interstitial wormshrimp, *Ingolfiella maldivensis*, is described from coral sand on the inner and outer reef off Magoodhoo island, Faafu atoll, Maldives. Six females were found and compared to other species from the Maldives and those bordering the Indian Ocean and beyond. Morphological resemblance ties it to a species from the Caribbean island of Curaçao. Both species are found in shallow sublittoral interstitial spaces.

## Introduction

The Maldive Islands (Central Indian Ocean) consist of a 800-km long string of 22 atolls containing an extensive coral reef system topped by over a thousand islands (Coleman 2000; Spalding et al. 2001). During field work off Magoodhoo island in the Faafu atoll, republic of Maldives, six female specimens of the rare amphipod family Ingolfiellidae were found. Ingolfiellids are known to live strictly subterranean in a wide variety of aquatic habitats; from the ocean floor to shallow marine interstitial sand habitats through to caves and brackish and fresh continental groundwater (Stock 1977; Vonk and Schram 2003).

The first Maldives Coral Reef Biodiversity Workshop located at MARHE Centre in Magoodhoo (May 2014) enabled sampling by use of SCUBA and access to a wide variety of suitable habitats for reef coral rubble inhabitants. Previously, only two other specimens of representatives of the family had been reported from the Maldives (Ruffo 1966).

Although their numbers are mostly low, the presence of vermiform and interstitial ingolfiellids or wormshrimps (Vonk and Nijman 2006) is expected for all tropical reef sand environments. As they have no free-swimming larvae in the water column and a low egg production (Siewing 1963) their capacity for long distance dispersal is presumably quite limited. Geographically separated populations show subtle but constant morphological differences and, in the absence of molecular phylogenetic comparisons, are considered to represent different species.

In this paper we describe *Ingolfiella maldivensis* sp. n. and discuss relations to other species.

## Material and methods

The six specimens of the new species were collected from two different sites (Fig. 5) by SCUBA diving between 2–25 m depth. A plastic probing tube of 12 cm and a diameter of 2.5 cm was drilled by hand into the sand at selected places were the top layer of coarse reef sand is thick enough as to allow vertical to slightly skewed probing. The top of the tube contains a small hole for escape of excess water. Then the tube is carefully removed, with the top closed and so creating a vacuum suction that prevents the sediment from falling out. After this a lid is quickly placed over the opening.

**Figure 1.**
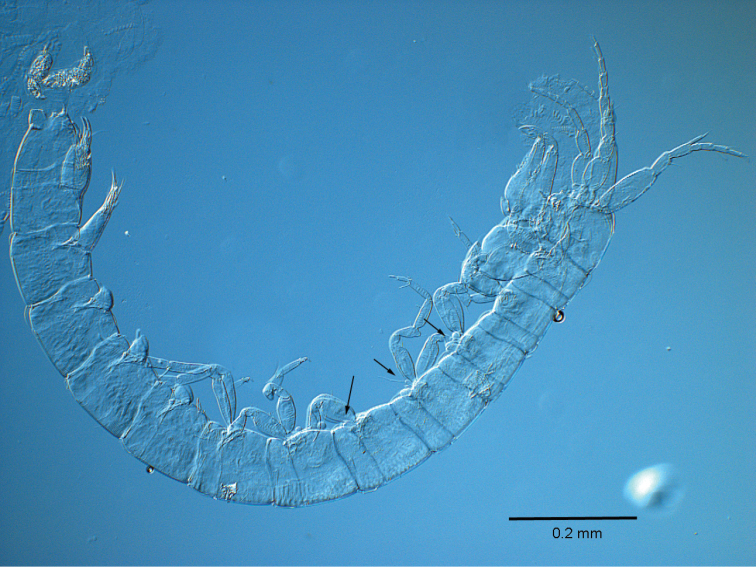
*Ingolfiella maldivensis* sp. n., holotype female 1.80 mm (including telescoped body somites). Arrows point to gills and oostegites on the third and fourth pereiopods, and on gills on the fifth pereiopod.

The samples were sorted in the Italian field station of the Milano-Bicocca Marine Research and High Education Centre (MARHE) under a dissecting microscope and transferred to 96% ethanol. Before study, specimens were treated with lactic acid to soften the cuticle and remove internal tissues to facilitate observation. Photo of entire animal (Fig. 1) was made with a Zeiss Axio Imager M2 microscope using differential interference contrast (DIC). Drawings were prepared using a camera lucida on an Olympus BX 53 microscope equipped with DIC. Specimens and appendages preserved on slides were mounted in Faure’s medium and the coverslips sealed with transparent nail varnish. Body measurements were derived from the sum of the maximum dorsal dimensions (including telescoped portions) of head, pereionites, pleosomites and urosomites, and exclude telson length. Following Watling (1989), the term “spine” in descriptions is restricted for rigid armature elements with a hollow central core that do not articulate basally to the body integument.

## Taxonomy

### Order AMPHIPODA Latreille, 1816 Suborder INGOLFIELLIDEA Hansen, 1903 Family INGOLFIELLIDAE Hansen, 1903 Genus *Ingolfiella* Hansen, 1903

#### Ingolfiella maldivensis sp. n.

Taxon classificationAnimaliaAmphipodaIngolfiellidae

http://zoobank.org/5B7816E3-73C4-4904-871B-AA12E5E0FDAD

Figs 1 , 2 , 3 , 4

##### Material examined.

One specimen, RMNH.CRUS.P.264, female paratype 1.85 mm, at station ‘Blu Cove’, 6 May 2014, depth 15 m, N3°05'37.8", E72°57'59.4". Five specimens: RMNH.CRUS.P.265, undissected female holotype 1.80 mm (Fig. 1); RMNH.CRUS.P.266, female paratype 1.55 mm; RMNH.CRUS.A.5054, female paratypes, at Dharamboodhoo reef, 11 May 2014, depth 20 m., N3°03'30.5", E72°55'29.6". All collected by R. Vonk. Specimens are stored in the Crustacea collection of Naturalis Biodiversity Center, Leiden.

##### Diagnosis.

Lateral lobes on frontal margin of the head present. Dactyls of gnathopods armed with four javelin lancet shaped bladelike spines along posterior margin. Palm of G2 angle robust seta bifid; posteromedial surface of carpus lacking broad triangular spine. Medial surface of protopod of U2 with three denticle combs. Unguis of P3–P4 with four denticles; that of P5–P7 bifid. PL1–PL3 present and of similar form. Oostegites on P3–P4.

##### Etymology.

The new species is named after the group of islands where it was found, in the Republic of the Maldives.

##### Description.

**Figure 2.**
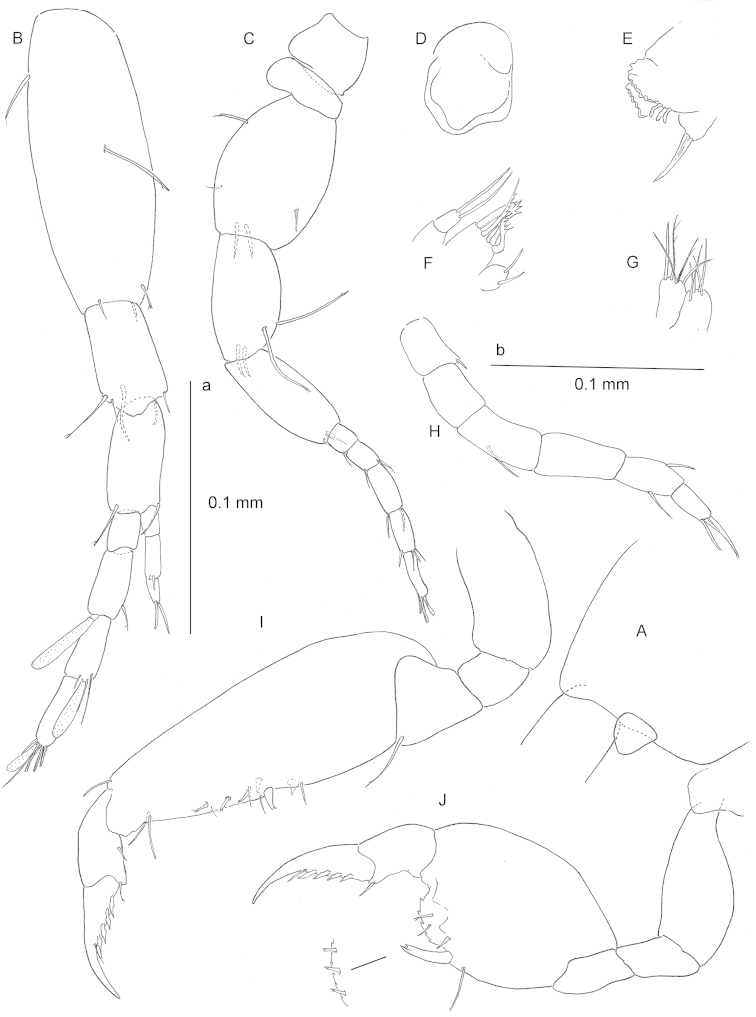
*Ingolfiella maldivensis* sp. n., holotype female 1.80 mm **A** cephalic lobe **B** antennule **C** paratype female 1.85 mm, antenna **D** paratype female 1.55 mm, labrum **E** right mandible with incisor and molar process **F** maxillule **G** maxilla **H** maxilliped **I** paratype 1.85 mm, right gnathopod 1, medial **J** right gnathopod 2, medial (inset: palm margin of holotype 1.80 mm).

Body elongate, cylindrical, without coloration, transparent to milky white (Fig. 1). *Head* with frontal margin nearly straight, no sinus visible, cephalic lobe placed a little backwards from the frontal margin (Fig. 2A).

*Antennule* (Fig. 2B), peduncle article 1 robust, slightly inflated, articles 2 and 3 of equal length. Flagellum 4-articulate, longer than peduncle articles 2–3 combined; proximal article unarmed and short, other three articles of equal length; articles 2–4 each provided with aesthetasc, aesthetascs progressively shorter towards distal. Accessory flagellum 3-articulate, shorter than two proximal articles of main flagellum combined.

*Antenna* (Fig. 2C) slightly shorter than antennule; gland cone short, hardly protruding dorsomedially; protopodal articles 3–5 inflated, especially the third one, fourth segment with two long setae on posterior margin; Flagellum 5-articulate, shorter than protopodal articles 4–5 combined.

*Labrum* (Fig. 2D) and paragnaths (not figured) ordinary, latter lacking inner lobes.

Mandibles with molar process non-triturative, spiniform and not serrated. *Right mandible* (Fig. 2E) with 6-denticulate incisor; spine row with three short, stubby, finely serrated elements. *Left mandible* (not figured) with spine row comprising two blunt finely ribbed elements.

*Maxillule* (Fig. 2F) coxal endite [= inner lobe] with two simple setae; basal endite [= outer lobe] with six robust setae of which two bicuspidate, one 3-cuspidate, one long and 5-cuspidate, one short, broad and 3-cuspidate, and one – the innermost – crooked and 4-cuspidate; endopod (=palp) 2-segmented, distal segment with two long slender setae.

*Maxilla* (Fig. 2G) with short, subequal blunt figs, outer one with five distal setae, inner one with four distal setae; two out of five setae on outer fig sparsely setulose.

*Maxilliped* (Fig. 2H) basal endite rudimentary, with one simple seta; merus with one simple seta on outer margin; propodus with two single simple seta on opposite margins; dactylus slender, with two distal setae, and long unguis.

**Figure 3.**
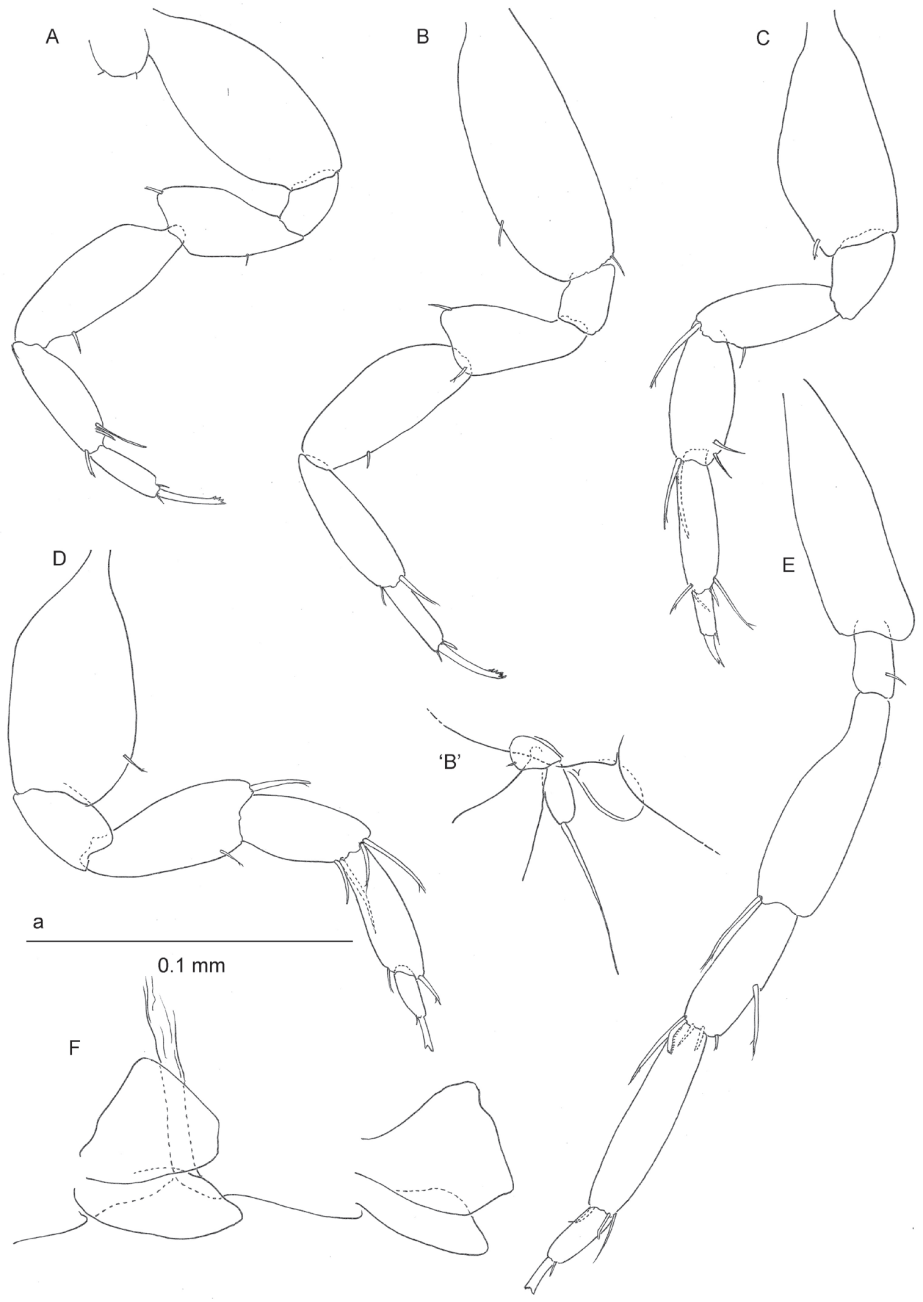
*Ingolfiella maldivensis* sp. n., paratype female 1.85 mm **A** pereiopod 3 **B** pereiopod 4 **B**’ holotype female 1.80 mm oostegite and gill on pereiopod 4 **C** paratype female 1.55 mm pereiopod 5 **D** paratype female 1.85 mm pereiopod 6 **E** pereiopod 7 **F** pleopods 2 and 3.

*Coxal gills* (Fig. 1) present on P3–P5, rounded to ovoid. *Oostegites* (Figs 1, 3B’) on P3–P4, short, subrectangular and shorter than corresponding coxal gill, each with one long slender seta.

*Gnathopod 1* (Fig. 2I) carpo-subchelate, carpus almost 3 times as long as broad and exceptionally slender and elongated toward the tip, with three short, bifid flagellate robust setae along lateral side of palm margin, one robust seta on palm angle, and one short stout simple seta and broad triangular spine on medial surface of segment as figured; palm margin slightly convex and smooth; dactylus with four slender stalked-lanceolate bladelike denticles along posterior margin.

*Gnathopod 2* (Fig. 2J) carpo-subchelate, carpus massive, shorter and broader than carpus of G1; palm margin strongly convex, clearly serrated, lined up with three short, bifid flagellate robust setae along lateral side; palm angle marked by stout, slightly curved bifid robust seta; medial surface of segment with short, simple robust seta that varies between individuals in width, however it is not a broad, strong triangular spine; posteromedial surface of carpus with excavation apparently to accommodate distal portion of unguis; dactylus with four lanceolate bladelike denticles along posterior margin.

*Pereiopods 3–4* (Fig. 3A, B) subequal except for slightly longer propodus in P4; dactylus elongate, with two simple setae at the base of the unguis; unguis slender and with four fine denticles on tip.

*Pereiopods 5–7* (Fig. 3C–E) progressively longer towards posterior; basis of P5–P6 broad, that of P7 slender. P7 with one of distal armature elements on distolateral angle of carpus modified into a crooked comb-like seta. Dactylus of P5–P6 short, that of P7 longer. Unguis of P5 bifid but not so outspoken as in P6–P7. Gill present on P5.

*Pleopods 1–3* (Fig. 3F) subtriangular.

**Figure 4.**
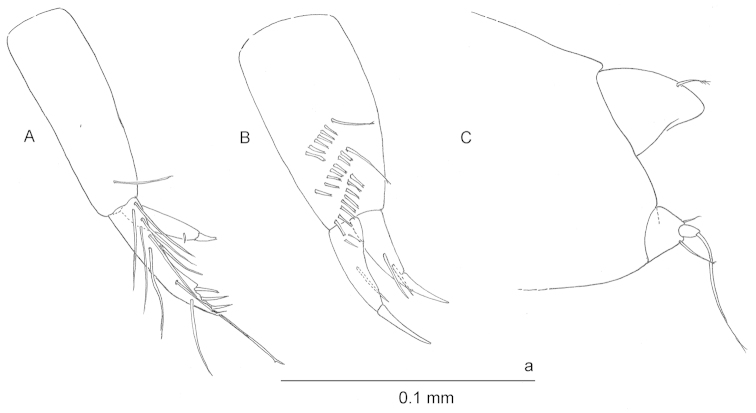
*Ingolfiella maldivensis* sp. n., paratype female 1.85 mm **A** uropod 1 **B** paratype female 1.55 mm uropod 2 **C** paratype female 1.85 mm uropod 3 and telson.

*Uropod 1* (Fig. 4A) protopod subrectangular; exopod much shorter than endopod, acuminate, with short robust seta terminally and tiny simple seta placed subdistally; endopod with short terminal spine plus row of three stout triangular robust setae subdistally; nine simple setae disposed on segment as figured.

*Uropod 2* (Fig. 4B) protopod bearing three oblique combs of mostly bifid spines on medial surface; two most proximal combs including one long seta; rami tapering, each with strong terminal simple seta clearly articulating at base, exopod stouter and slightly shorter than endopod.

*Uropod 3* (Fig. 4C) small and uniramous, protopod triangular, with two setae flanking the short exopod; exopod with long apical seta.

*Telson* (Fig. 4C) entire and thick, fleshy, with one plumose seta distomarginally at each side.

##### Remarks.

Previous knowledge on the ingolfiellids from the Maldives was restricted to specimens collected during the Xarifa Expedition 1957–1958. These came from washings of the coral *Favites* sp. (Ruffo 1966). They were described as *Ingolfiella xarifae* Ruffo, 1966 and came from Rasdu atoll, some 130 kilometers north of the Faafu atoll where specimens of the present species were found. *Ingolfiella xarifae* differs from the rest of *Ingolfiella* species by having three denticles on the posterior margin of the dactylus of the first gnathopod, and four on the second (see Vonk and Jaume 2014). They also have a trifid unguis on the third and fourth pereiopod, and a broad triangular spine on the posteromedial surface of the carpus in the second gnathopod. These features set them clearly apart from *Ingolfiella maldivensis* sp. n. which has four denticles on both gnathopod dactyli, multidenticulate claws and no triangular spine on the carpus of the second gnathopod.

A comparison of fourteen easy to distinguish characters between members of the genus *Ingolfiella* (45 species) was done by Vonk and Jaume (2014). The new species ranks next to a Caribbean species from comparable shallow sublittoral habitats off the coast of Curaçao, namely *Ingolfiella quadridentata* Stock, 1979. Character states overlap entirely for the eight non-male-specific features of Vonk & Jaume’s matrix, but other features differ. Thus, the basal endite of the maxilliped is small and barely developed in *Ingolfiella maldivensis*, but separate and as long as the ischium in *Ingolfiella quadridentata*; the triangular spine on the posteromedial surface of carpus of the first gnathopod is lacking in *Ingolfiella quadridentata*; the oostegites are crowned with one long seta in *Ingolfiella maldivensis*, but have a 3-pronged distal margin in *Ingolfiella quadridentata*; and the claw of the fourth pereiopod is 4-denticulate in *Ingolfiella maldivensis* but 7-denticulate in *Ingolfiella quadridentata*.

Other species bordering the Indian Ocean include: *Ingolfiella kapuri* Coineau & Rao, 1973, from the Andaman and Nicobar Islands in intertidal shell debris; *Ingolfiella arganoi* Iannilli & Vonk, 2013 from Abd-al-Kuri Island, Socotra Archipelago in an anchialine pool; *Ingolfiella quokka* Gallego-Martínez & Poore, 2003, from an intertidal sandy beach environment from the City of York Bay, Western Australia. All of these species differ sharply from *Ingolfiella maldivensis* (see Vonk and Jaume 2014: table 1).

The recently described *Ingolfiella botoi* Vonk & Jaume, 2014, from beach groundwater in the Gura Ici Islands, Molucca Sea, Indonesia (Vonk and Jaume 2014), shares more features with *Ingolfiella maldivensis* than with the rest of the Indian Ocean species mentioned above. It can be remarked that the Maldives, forming the western rim, and the Moluccas, positioned in the middle, are both still part of one large Indo-Polynesian marine biogeographic province (Briggs and Bowen 2012).

## Discussion

Repeated visits to the same island groups or to mainland karst areas have often revealed additional species each time a specific search for ingolfiellids was made. In other cases populations of the same species are spread over different islands. This was encountered in the Canary islands for the widely separated islands Hierro and Tenerife (Vonk and Sánchez 1991; Vonk and Jaume 2014). Yet also in that same Canary island group two other, different, species where found: *Ingolfiella similis* on Fuerteventura (Rondé-Broekhuizen and Stock 1987) and *Ingolfiella* sp. on Lanzarote (Wilkens et al. 2009). On the Philippines *Ingolfiella alba* appears in littoral sands of more than one island (Iannilli et al. 2008) and remains the only species known from that large archipelago. But in the small Indonesian Gura Ici island group in the Molucca Sea two species appear in syntopy in the same beach groundwater spot (Vonk and Jaume 2014). After many years of sampling in the Caribbean islands of Aruba, Curacao, and Bonaire five species in diverse aquatic habitats such as marine sublittoral carbonate sands, brackish caves and terrestrial groundwater were recognized (Stock 1979).

These examples lead to the expectation that an ocean spanning, circumtropical continuum exists of populations gradually changing in minor morphological adaptations and converging in functional form toward their environment. Such convergence could explain why a sublittoral reef sand inhabiting form from the Caribbean is more similar to a form that lives in comparable micro-habitats in the Indian Ocean, than it is to a congener (*Ingolfiella grandispina* Stock, 1979) found a few kilometers away in a brackish cave bottom with other functional requirements to form. This convergent development can be observed in the close morphological resemblance of *Ingolfiella quadridentata* from the Caribbean island of Curacao and the species, described in this study, *Ingolfiella maldivensis*. They both come from sublittoral reef sands.

The Maldives have undergone dramatic sealevel changes (Aubert and Droxler 1992; Gischler et al. 2014). This has changed the islands from karstic, well emerged platforms with ample subterranean habitat types to the flat atolls of today (Schlager and Purkis 2013). Future discoveries of relicts of this subterranean diversification may reflect this geological past.

**Figure 5.**
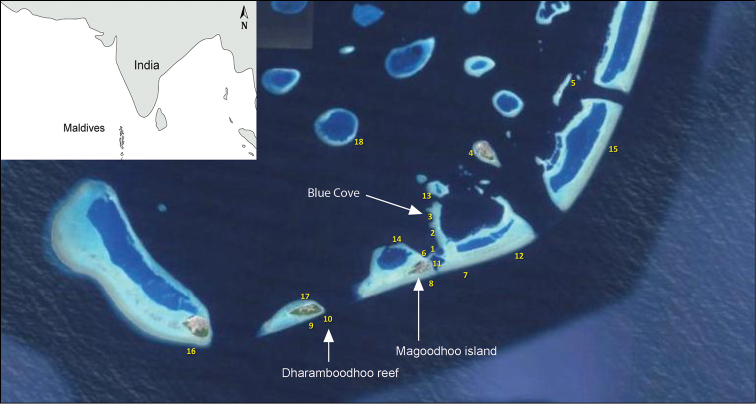
Map of dive sites around Magoodhoo island, Faafu atoll, Republic of the Maldives (Modified from Montano et al. 2014). Stations 3 (inner reef) and 10 (outer reef) contained ingolfiellid amphipods.

## Supplementary Material

XML Treatment for Ingolfiella maldivensis
